# Neural Representation of Motor Output, Context and Behavioral Adaptation in Rat Medial Prefrontal Cortex During Learned Behavior

**DOI:** 10.3389/fncir.2018.00075

**Published:** 2018-10-01

**Authors:** Roel de Haan, Judith Lim, Sven A. van der Burg, Anton W. Pieneman, Vinod Nigade, Huibert D. Mansvelder, Christiaan P. J. de Kock

**Affiliations:** Center for Neurogenomics and Cognitive Research, Department of Integrative Neurophysiology, VU Amsterdam, Amsterdam, Netherlands

**Keywords:** medial prefrontal cortex, tactile decision making, behavioral adaptation, mPFC, electrophysiology, spiking modulation

## Abstract

Selecting behavioral outputs in a dynamic environment is the outcome of integrating multiple information streams and weighing possible action outcomes with their value. Integration depends on the medial prefrontal cortex (mPFC), but how mPFC neurons encode information necessary for appropriate behavioral adaptation is poorly understood. To identify spiking patterns of mPFC during learned behavior, we extracellularly recorded neuronal action potential firing in the mPFC of rats performing a whisker-based “Go”/“No-go” object localization task. First, we identify three functional groups of neurons, which show different degrees of spiking modulation during task performance. One group increased spiking activity during correct “Go” behavior (positively modulated), the second group decreased spiking (negatively modulated) and one group did not change spiking. Second, the relative change in spiking was context-dependent and largest when motor output had contextual value. Third, the negatively modulated population spiked more when rats updated behavior following an error compared to trials without integration of error information. Finally, insufficient spiking in the positively modulated population predicted erroneous behavior under dynamic “No-go” conditions. Thus, mPFC neuronal populations with opposite spike modulation characteristics differentially encode context and behavioral updating and enable flexible integration of error corrections in future actions.

## Introduction

The medial prefrontal cortex (mPFC) integrates and processes a multitude of information streams to drive behavior (Groenewegen and Uylings, 2000; Dalley et al., 2004; Gruber et al., 2010; Euston et al., 2012; Luchicchi et al., 2016). Activity of mPFC neurons correlates with task outcomes with both positive (Gruber et al., 2010; Horst and Laubach, 2013; Orsini et al., 2015; Pinto and Dan, 2015; Amarante et al., 2017) and negative valence (Senn et al., 2014; Halladay and Blair, 2015; Pinto and Dan, 2015; Kim et al., 2017; Rozeske et al., 2018) and distinct populations of mPFC neurons are selectively activated during movement and movement inhibition (Halladay and Blair, 2015). Moreover, incorporation of task rules in the mPFC leads to adaptive strategies to optimize task outcome (Durstewitz et al., 2010; Euston et al., 2012; Horst and Laubach, 2012; Narayanan et al., 2013; Cho et al., 2015; Orsini et al., 2015; Guise and Shapiro, 2017; Malagon-Vina et al., 2018). Additionally, the mPFC is critically involved in bottom-up detection of tactile sensory input (Le Merre et al., 2018), as well as top-down filtering of visual and auditory sensory information (Zhang et al., 2014; Wimmer et al., 2015, 2016; Kim H. et al., 2016; Schmitt et al., 2017). Not surprisingly, neuronal correlates of sensory information are found in the mPFC during auditory “Go”/“No-go” tasks (Pinto and Dan, 2015; Kamigaki and Dan, 2017) and during a visual attention task (Kim H. et al., 2016). Furthermore, short-term task rules are represented transiently by spiking patterns in mPFC populations (Durstewitz et al., 2010; Rodgers and DeWeese, 2014; Malagon-Vina et al., 2018) and combined audiovisual selective attention tasks require correct spiking in mPFC axons to thalamus (Wimmer et al., 2015; Schmitt et al., 2017). The mPFC is thus ideally situated in the circuitry to drive learned behavior under conditions when sensory information guides decision making and behavioral output. As a result, perturbation of normal mPFC function leads to a diversity of behavioral impairments (Narayanan and Laubach, 2006, 2008; Pinto and Dan, 2015; Koike et al., 2016; Lagler et al., 2016; Luchicchi et al., 2016; Bolkan et al., 2017; Guise and Shapiro, 2017).

Motor behavior also shows neurophysiological correlates in the mPFC (Horst and Laubach, 2013; Pinto and Dan, 2015; Amarante et al., 2017) and disturbing the mPFC network can lead to reduced attention performance and inappropriate motor output (Narayanan and Laubach, 2006; Narayanan et al., 2006; Luchicchi et al., 2016; Kamigaki and Dan, 2017). However, it is currently unknown how the mPFC controls and encodes this motor behavior or whether it encodes just a copy of the motor signal (efference copy). Similarly, we know that inhibitory control and top-down increase of stimulus discrimination depend on mPFC function (Zhang et al., 2014; Pinto and Dan, 2015; Wimmer et al., 2015), yet we do not know how these are encoded. The executive function of the mPFC in adaptive behavior occurs on short timescales implying that the mPFC should integrate trial outcomes and adapt behavioral strategies on a trial-by-trial basis (Narayanan and Laubach, 2008; Euston et al., 2012; Horst and Laubach, 2012; Narayanan et al., 2013; Pinto and Dan, 2015), nevertheless we do not know how these are represented by mPFC neuronal spiking.

To identify neurophysiological correlates of sensory-guided and adaptive motor behavior, we recorded activity of mPFC neurons extracellularly (Csicsvari et al., 2003; Rossant et al., 2016) in adult rats performing a whisker-based “Go”/“No-go” object localization task (O’Connor et al., 2010a; Pammer et al., 2013). We present four findings on populations of putative pyramidal mPFC neurons, which show modulation of their spike rates: (1) during correct performance of the task; (2) during motor output with and without the intent to collect reward; (3) when changing behavior after mistakes; and (4) as a function of increasing task difficulty.

## Materials and Methods

### Animal Welfare Statement

This study was carried out in accordance with European and Dutch law and approved by the animal ethical care committee of the VU Amsterdam and VU University Medical Center, Netherlands (protocol INF-14-08).

### Surgery

Male Wistar rats (250–350 g, 8–12 weeks) were implanted with a headpost. Preoperatively Baytril (5 mg/kg), Temgesic (buprenorphine 0.05 mg/kg) and carprofen (5 mg/kg) were administered subcutaneously. Animals were anesthetized with 2% isoflurane for induction, placed on a heating mat and fixed into a stereotactic frame, after which the isoflurane concentration was reduced to 1.5% for maintenance. The hair on the head was removed with hair clippers and lidocaine was injected for local analgesia (200 μl, 2% *s.c*.). An incision was made in the skin, the underlying tissue was removed and the skull cleaned. Next, the skull was etched with Gel Etchant (Kerr Dental, Visé, Belgium) and cleaned. Bonding agent (Optibond, Kerr Dental, Visé, Belgium) was used to enhance adhesion between dental cement (Tetric evoflow, Ivoclar Vivadent, Schaan, Liechtenstein) and the bone. Four screw holes were drilled (1× occipital bone, 1× contralateral parietal bone (above V1), 1× frontal bone (above the olfactory bulb) and 1x lateral of the temporal ridge) and stainless steel head screws were inserted to increase stability of the head cap. The craniotomy for electrophysiological mPFC recordings was drilled at 2.5 mm anterior and 0–1.2 mm lateral of bregma and recordings were targeted to the ventral anterior cingulate (AC), prelimbic (PL) and dorsal infralimbic (IL) cortex (at 1,900–3,500 μm from pia on the dorso-ventral axis).

### Behavioral Task: Whisker Based Head-Fixed “Go”/“No-Go” Task

**Figure 1:**
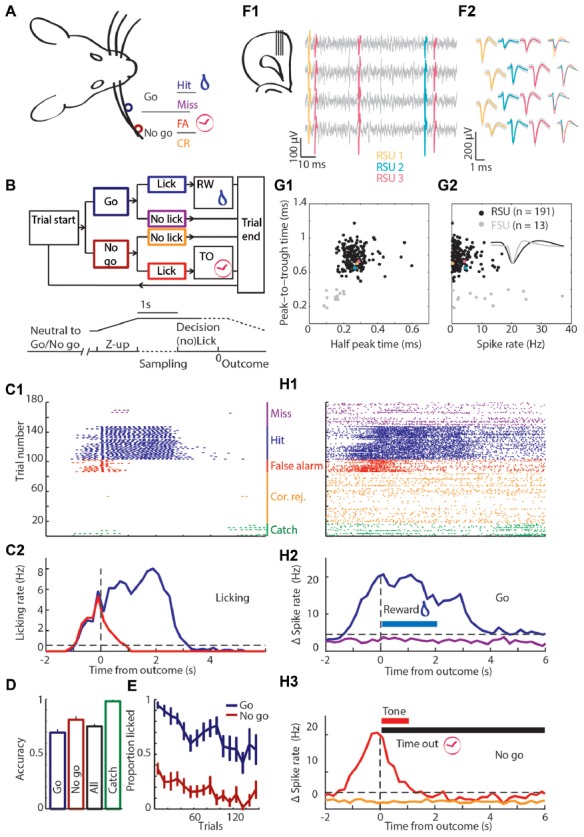
Experimental approach. **(A)** Schematic of the head-fixed rat. Licking when the object was in the “Go” position (dark blue) was rewarded with water and called a Hit trial (blue), while refraining from licking was called a Miss trial (purple). When the object was in the “No-go” position (brown-red) the rat should refrain from licking to make a Correct Rejection (CR; orange), while licking in this condition was a False Alarm (FA; red) and punished with a tone and time out (TO). **(B)** Block diagram showing the sequence of actions during trials. The object moved from a position in the middle between “Go” and “No-go”, while out of reach of the whiskers. Subsequently, the pole moves up, the rat was allowed to lick during the sampling period (1 s), but licking was neither punished nor rewarded. During the 1 s decision window licking triggers an outcome (rewarded or punished for “Go” or “No-go”, respectively). Colors as in **(A)**. **(C)** The licking behavior of a rat during a single behavioral session. **(C1)** Licking raster plot of the 180 trials that were performed during this session, sorted by trial outcome and color coded as in **(A)**. Every tick is a lick that was counted as a beam break in the lick port. We intermixed 10% Catch trials without the object (green). **(C2)** Peri-event time histogram (PETH) of licking around trial outcome (first lick during the decision window) for Hit and FA trials. **(D)** Average accuracy over all rats. **(E)** Licking probability over time for all sessions. **(F1)** Cartoon of the probe placement (left) and in gray four example channels with spikes of three units superimposed in color. **(F2)** The average waveform of the units that are shown in **(F1)** on the same four channels. **(G1)** Regular spiking units (RSUs; black) and fast spiking units (FSUs; gray) were separated based on their average waveforms. Colored dots are the spikes from **(F1,F2)**. **(G2)** The FSUs show a wider distribution of spike rates. Inset are example FSU and RSU waveforms. **(H1)** Spiking raster plot of an example unit. Same session, sorting and color code as in **(C1,C2)**. **(H2)** PETH of spiking of the unit from **(H1)** around “Go” trial outcomes with baseline subtracted. Horizontal blue bar is the window during which water is available from the lick port. **(H3)** As **(H2)** for “No-go” trials. Horizontal red bar is the window in which the tone is broadcast, the horizontal black bar shows the duration of the TO.

To study the neural basis of cognitive behavior in the mPFC, we adapted a whisker-based object localization (“Go”/“No-go”) task for head-fixed rats. The task was adapted for rats from similar tasks in mice (O’Connor et al., 2010a; Pammer et al., 2013) and development during the start-up phase was facilitated by expert input from Dr. Karel Svoboda (HHMI, Janelia farm, Ashburn, VA, USA) and Dr. Cornelius Schwarz (CIN, Tübingen, Germany). To drive motivation for optimal task performance, the rats were maintained on temporary water restriction such that they earned all their water by performing the task (weekly schedule of 5 days training, 2 days *ad libitum* access). Rats learned to distinguish two locations of a cue-pole and were rewarded when responding with motor output (i.e., licking) to the “Go” location. To promote discrimination and avoid simple detection strategies (e.g., keeping whiskers on one of the two locations), both locations were placed on the same azimuth of the resting position of the C1 whisker, but were spaced 4 mm on the proximal-distal axis (Figure 1A). During behavioral training, rats learn to lick for a reward when the object was in the proximal (“Go”) position and refrain from licking in the distal (“No-go”) position. To monitor the rats’ health and water intake, both daily water volume consumed and the weight of the rat were closely monitored and behavioral abnormalities were registered. When the rat did not drink enough during training and consequently lost weight, a small volume (typically 2–4 ml) of water was offered to the rat.

### Training Regime

Trials started with the pole outside the reach of the whiskers in a neutral position, exactly in the middle of the “Go” and “No-go” positions to reduce detectability of the pole location by cues other than whisker touch (e.g., timing or sound cues). Trial identity varied randomly, but consecutive trials were limited to four of the same “Go”/“No-go” identity. After trials started, the object moved to either the “Go” or “No-go” position. The pole then moved up into the whisker field by valves driven by air pressure, which produced a soft clicking sound upon action. After the pole was fully up, a 1 s grace period started for sensory acquisition during which licking was allowed, but not rewarded or punished. Rats repeatedly touched the object with their whiskers during both “Go” and “No-go” trials (Supplementary Figure S1). Following the grace period, the rat had 1 s to lick if it decided the pole was in the “Go” position. Licking was recorded by beam breaks of an infrared laser beam in the lick port (Sunx, West Des Moines, IA, USA). When rats licked during “Go” trials, the trial was labeled a Hit trial and a 20–40 μl water was given (two drops separated in time by 1 s). When rats licked during “No-go” trials, the trial was labeled a False Alarm (FA) trial and the mistake was signaled with a 1 s 12 kHz sound at 70 dB followed by a time out (TO) period (5–7 s depending on previous session “No-go” outcome). In addition, the probability of another “No-go” trial was increased after FA errors to discourage “always-lick” strategies (fraction “Go” trials, 0.25). When the rat did not lick in “Go” or “No-go” trials, the trial outcome was labeled as Miss or Correct Rejection (CR), respectively. Trials without licking were not rewarded nor punished (Figure 1B) and all trials ended with the pole moving back to the neutral position. We used a variable inter-trial interval period of 1.5, 2 or 3 s to reduce predictability of task-timing. Rats were trained until they reached performance accuracy above 70% for “Go” and “No-go” trials collectively ((Hit + CR)/(“Go” + “No-go” trials) * 100%). Whisker object-touches were comparable between “Go” and “No-go” trials (Supplementary Figure S1). To confirm that the rats use whisker-guided sensory cues for decision making, we intermixed 10% catch trials (no object) in which the valves to control the pole z-position made similar sounds, but the pole did not move within reach of the whiskers. Licks were not rewarded or punished during Catch trials and rats usually did not lick during Catch trials.

After reaching threshold for task performance, the rats daily performed three types of sessions in random order. Apart from the regular session type, we changed the task in a second session type such that the “No-go” location was randomly selected from an Easy, Normal or Hard position (2, 4 and 8 mm from the “Go” position, respectively; Figure 6A). This manipulation allowed us to test mPFC physiological dependence on changes of stimulus contrast. In the third session type we changed the reward or punishment during random trials: in a subset of Hit trials, we doubled the water reward to investigate scaling of reward related spiking with reward magnitude. In a different subset of Hit trials, we delayed the delivery of the reward with 1 s to distinguish between the neurophysiological correlate of simple licking-induced motor activity and actual reward feedback and consumption. In the same session type we used trials in which the punishment signal was delayed with 1 s.

**Figure 2:**
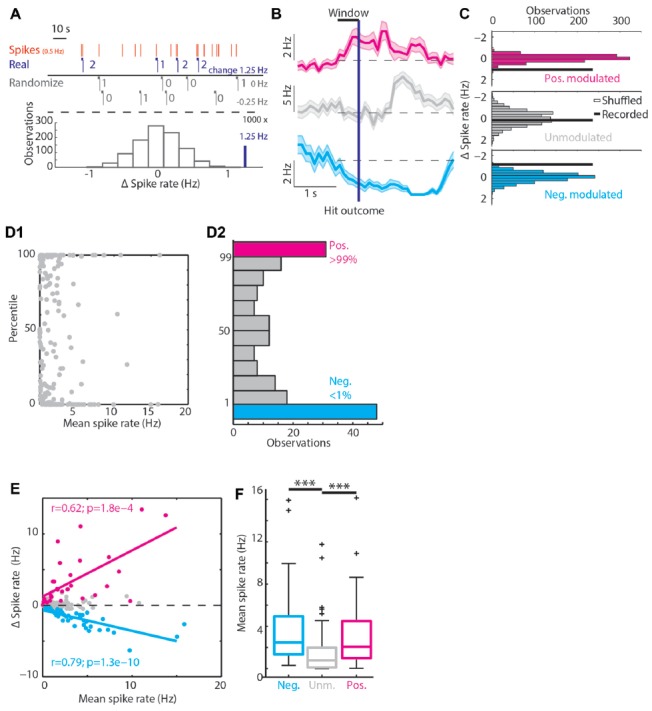
Classifying RSUs based on Hit trial spike rate modulations. **(A)** A simulated example of the bootstrapping method. In red are the spikes of a unit spiking on average with 0.5 Hz. The horizontal blue bars show the 1 s windows before Hit outcomes, the blue numbers give the number of spikes in each window. In black we show two randomizations of the Hit time stamps and the corresponding Δ spike rate. Under the dashed line we show in black a histogram of Δ spike rates (black) and the recorded Δ spike rate (blue). In this case the recorded Δ spike rate was in the 99th percentile of the randomized distribution and is considered positively modulated during Hit trials. **(B)** The PETHs of three example units around Hit outcomes. **(C)** The histograms of the bootstrapped spike rate distribution and the recorded Δ spike rate as a thick black line. Same units as in **(B)**. The magenta unit was positively modulated, the gray unit unmodulated and the cyan unit was negatively modulated. **(D1)** The distribution of percentiles from the bootstrapping method with respect to the mean spike rate during the whole session. **(D2)** Histogram over the percentile axis of **(D1)**. **(E)** Correlations between mean spike rates and Δ spike rate. Units that have a high spike rate will show the largest Δ spike rate, positive (magenta) or negative (cyan). **(F)** The positively and negatively modulated population have a significantly higher median spike rate compared to the unmodulated units. ***p < 0.001.

**Figure 3:**
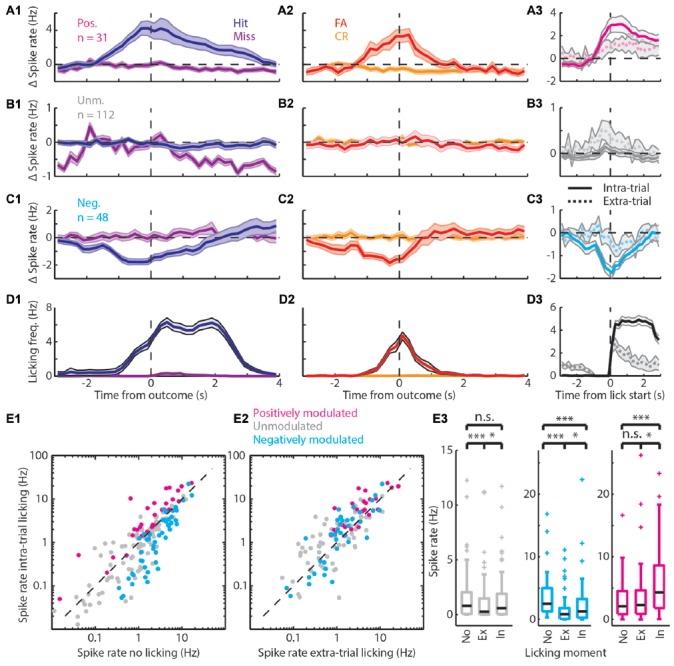
Spiking modulation encodes motivation in addition to motor output. **(A)** Average PETHs of spiking of the positively modulated population (*n* = 31). **(A1)** Spiking aligned to “Go” trial outcome. **(A2)** As in **(A1)** but for “No-go” trials. **(A3)** Spiking aligned to lick start for intra-trial licking bouts (solid line) and extra-trial licking bouts (dashed line). **(B) (B1–B3)** analogous to **(A1–A3)** but with PETHs of the unmodulated population (*n* = 112). **(C) (C1–C3)** analogous to **(A1–A3)** but with PETHs of the negatively modulated population (*n* = 48). **(D) (D1–D3)** analogous to **(A1–A3)** but with licking PETHs. See Supplementary Figure S1 for a separation of units that are (un)correlated to licking frequency. **(E1)** Scatterplot of spike rate outside licking bouts vs. spike rate during intra-trial licking bouts. Note the shift from the unity line for the positively (magenta) and negatively (cyan) modulated units. **(E2)** Scatterplot of spike rate during extra-trial licking bouts vs. intra-trial licking bouts. Note the shift of both the positively and negatively modulated populations above the unity line. **(E3)** Boxplots of spike rates outside of licking bouts (No), during extra-trial licking bouts (Ex) and during intra-trial licking bouts (In) for the three functional populations. n.s. not significant (*p* > 0.05), *p < 0.05, ***p < 0.001.

**Figure 4:**
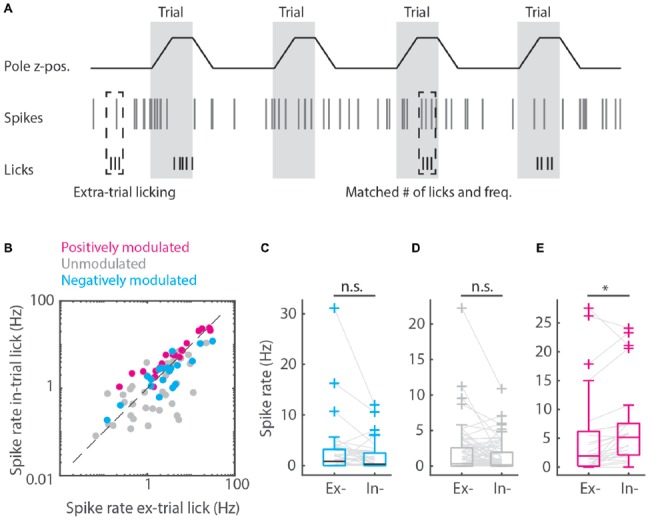
Matched extra-trial and intra-trial licks reveals the contribution of context to spike rate modulation. **(A)** Schematic representation of the procedure of matching behavioral statistics in which extra-trial licking bouts are matched with intra-trial licking bouts based on comparable number of licks and licking frequency to parse out the contribution of context to spike rate modulation. **(B)** Scatterplot of spike rates during intra-trial licking bouts vs. spike rates during extra-trial licking bouts for the positively modulated (magenta), unmodulated (gray) and negatively (cyan) modulated populations. **(C,D)** The negatively modulated population **(C)** and unmodulated population **(D)** did not show spiking rate modulations for matched intra- and extra-trial licking bouts, indicating that context is not involved in spike rate modulation for these populations. **(E)** The spiking rate of the positively modulated population was significantly higher during intra-trial than during extra-trial licking bouts, which is supportive evidence for the hypothesis that context is an important contributor to spike rate modulation for this population of medial prefrontal cortex (mPFC) units. n.s. not significant (*p* > 0.05), *p < 0.05.

**Figure 5:**
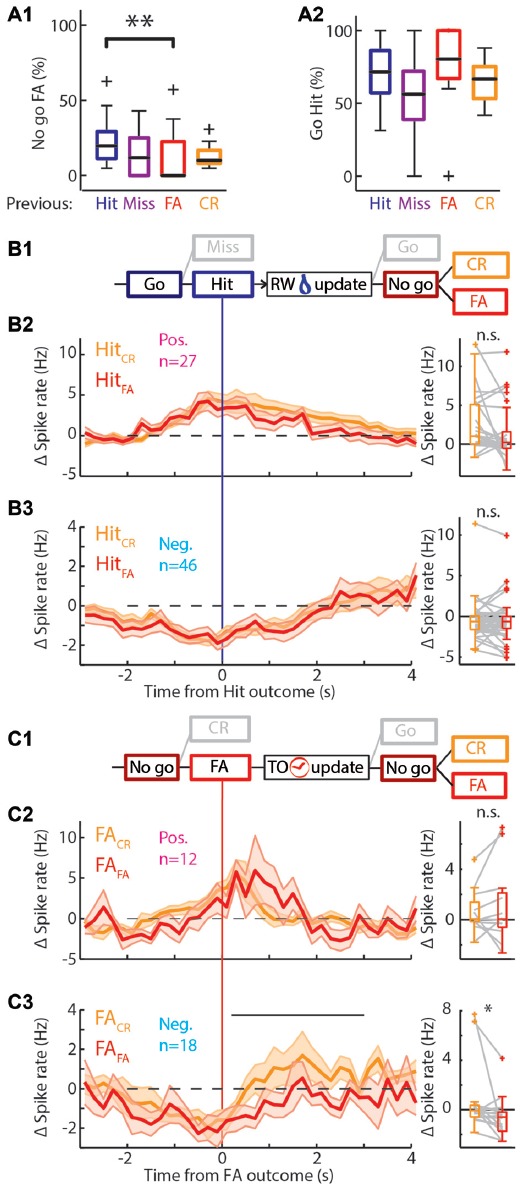
Behavioral change upon feedback. **(A)** Boxplots of the performance of the rat separated by outcome of the previous trial. **(A1)** There are significantly more/fewer FA errors in “No-go” trials if the previous trial was a Hit/FA respectively. **(A2)** There was no significant difference in the performance in “Go” trials based on the previous trial outcome. **(B1)** Schematic representation of the selection of trials for **(B2,B3)**. Only those Hit trials that are followed by a “No-go” trial are used. RW is water reward. **(B2)** Left: average PETHs of the positively modulated population around Hit outcomes for trials that were followed by a “No-go” trial. Hit trials that were followed by CR (Hit_CR_; orange) and those that were followed by a FA (Hit_FA_; red) show similar time courses of Δ spike rate (*n* = 27). Right: there was no significant difference of the median Δ spike rate in Hit_CR_ and Hit_FA_ trials. **(B3)** As **(B2)** for the negatively modulated population (*n* = 46). **(C1)** Similar to **(B1)** for FA trials. TO is time out punishment. **(C2)** Left: PETHs of the Δ spike rate of the positively modulated population during FA trials that are followed by a CR trial (FA_CR_; orange) and those that are followed by another FA trial (FA_FA_; red; *n* = 12). Right: there was no significant difference of the median Δ spike rate in FA_CR_ and FA_FA_ trials. **(C3)** As **(C2)** for the negatively modulated population (*n* = 18). The black bar indicates the window for right panel. Right: the median Δ spike rate during 0.2–3 s after the FA trial outcomes was significantly higher during FA_CR_ trials than FA_FA_ trials. n.s. not significant (*p* > 0.05), *p < 0.05, **p < 0.01.

**Figure 6:**
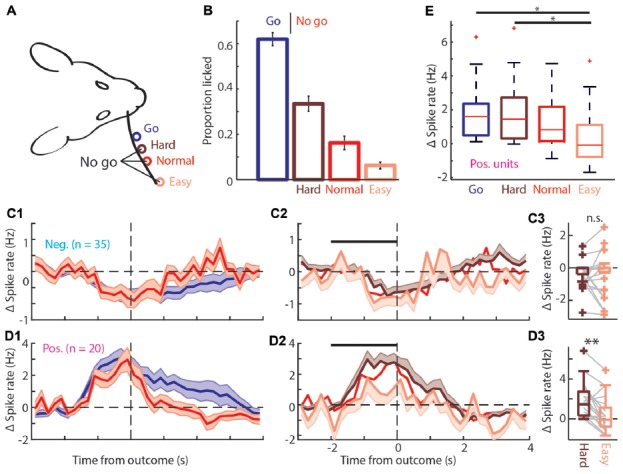
The positively modulated units have to be sufficiently excited for correct performance in easy “No-go” trials. **(A)** Schematic of the head-fixed rat and corresponding “Go” (blue), and three types of “No-go” trials, Hard (brown), Normal (red) and Easy (coral). **(B)** Proportion of trials licked during “Go” and the three types of “No-go” trials. The rats are most likely to lick during “Go” trials and the licking probability decreases progressively for Hard, Normal and Easy “No-go” trials. **(C1)** Average PETHs of spiking during “Go” and Normal “No-go” trials for the negatively modulated population (*n* = 35). The time course of both trial types was similar to the spiking recorded in the regular session (see Figures 3C1,C2). **(C2)** Average PETHs of spiking for the three types of FA trials. Black bar is window for **(C3)**. **(C3)** Boxplot of Δ spike rates of the negatively modulated population during the −2 to 0 s from the outcome for Hard and Easy FA trials. **(D1)** As in **(C1)** for the positively modulated population (*n* = 20). **(D2)** As in **(C2)** for the positively modulated population. **(D3)** As in **(C3)** for the positively modulated units. The median Δ spike rate was significantly higher for Hard trials than for Easy FA trials. **(E)** Boxplot for the Δ spike rate of the positively modulated population −2 to 0 from trial outcome (first lick in the decision window) for the four licking trials (Hit and Hard, Normal and Easy FA trials). The Δ spike rate was significantly lower during Easy FA trials than Hit and Hard “No-go” trials.n.s. not significant (*p* > 0.05), *p < 0.05, **p < 0.01.

### Electrophysiology

We used the Open Ephys data acquisition board with two RHD2132 digital interface chips (Intan Technologies, Los Angeles, CA, USA) and the open ephys GUI (Siegle et al., 2017) to record the electrophysiological activity of the mPFC. We used 4-shank 64-channel silicon probes (Cambridge Neurotech, Cambridge, UK). On these probes, 16 channels were clustered per shank in two parallel columns so that each channel has a distance of 25 μm to its neighbors. The shanks were spaced at 250 μm and were placed on the lateral-medial axis, i.e., throughout the layers of the mPFC. We saved the data as 16-bit integers at 30 kHz. Afterwards, we high-pass filtered the data and combined the 16 channels of each shank for automated clustering using Klustakwik (Rossant et al., 2016). We manually curated the clusters to get stable and well-isolated single units. We used stringent cut-offs; a unit was considered well-isolated if it had an isolation distance (ID) > 40, L-ratio <1, the cluster exceeded 300 spikes and the fraction of interspike intervals below 1.5 ms was <0.1%. This resulted in an average yield of 0.2 unit/recording site. Next, analysis was done on the average waveforms of all well-isolated units to further sub-classify units as regular spiking units (RSUs) vs. fast spiking units (FSUs; Barthó et al., 2004). Since FSUs (AP peak-to-trough time <0.5 ms and AP half-peak time <0.25 ms) represent a vastly heterogeneous population with a broad spectrum of functional and morphological characteristics, units with fast spiking waveforms were excluded from further analyses. Data obtained from three rats passed behavioral and electrophysiological criteria for subsequent data analysis.

Two distinct methods to place the silicon probe were used. In two rats we implanted the silicon probe on a nano-drive (Cambridge Neurotech, Cambridge, UK) with the electrodes in the dorsal mPFC. After the rat was trained we moved the probe down through the dorsal-ventral extent of the mPFC to record at multiple locations while rats were performing the behavioral task. In one rat, we positioned an acute silicon probe (same lay-out as the chronic probes) in the mPFC with a Luigs and Neumann manipulator (Ratingen, Germany).

### Perfusion, Slicing and Histology

After data acquisition was completed, rats were deeply anesthetized with 3% isoflurane and urethane (i.p. 10 mL/kg 20%). Next, rats were perfused transcardially with 0.9% NaCl followed by 4% paraformaldehyde (PFA). Brains were extracted and stored overnight in 4% PFA in phosphate buffer (PB) at 4°C after which they were transferred to 0.05 M PB for further processing.

Brains were sliced into 100 or 50 μm coronal sections with a vibratome, rinsed with PB (0.05 BM) and mounted using a Mowiol solution (Clairant GmbH, Frankfurt am Main, Germany; Narayanan et al., 2014). Probe placement was verified visually using an Olympus BX51 microscope with a 4× air objective or 40× oil objective. For chronic recordings, we used the electrode tract to retrieve probe placement. For acute recordings, we dipped the probe in DiI (Thermo Fisher Scientific, Waltham, MA, USA) and used a X-Cite 120 Q light-source (Excelitas Technologies Corp., Waltham, MA, USA), to visualize the electrode tract.

### Behavioral Quantification

Performance was quantified either as accuracy (number of correct over total number of trials of a type; Figure 1D), or as proportion of trials with licking in the response window for both “Go” and “No-go” trials (Figures 1E, 5A, 6B). Each lick was defined as the first time the infrared beam in the lick port was broken if it had remained unbroken for at least 10 ms. Licking Peri-event time histograms (PETHs) were made by binning licking time-stamps in 200 ms bins and averaging the detected licks per trial type.

To distinguish between spiking during motivated licking for a reward or during randomly emitted licks, spiking profiles for intra-trial vs. extra-trial licking bouts were computed. A licking bout was defined as the time between the first lick and last lick if all inter-lick-intervals in between were less than 1 s. We then defined a licking bout to be intra-trial if it started between 2 s before to 0.2 s after the outcome of a trial and extra-trial if it was outside of these windows. We then computed PETHs with 200 ms bins and from −3 s to +3 s from lick bout start for both the intra-trial and extra-trial licking bouts (spiking in Figures 3A3–C3, licking in Figure 3D3).

Quantification of spike rates per licking condition (i.e., no lick, intra-trial and extra-trial lick bouts) was done per unit as an average during all lick bouts (or all non-lick periods, Figure 3E). The distinction between intra- and extra-trial licking was made to have a measure for motor control (extra-trial licking) vs. a measure for the mixture of sensory, motivational, reward-driven and motor activity (intra-trial licking). To rule out that differences in licking behavior during intra- vs. extra-trial licking bouts would underlie differences in spiking between conditions, we matched individual extra-trial licking bouts based on equal number of licks and a licking frequency (# licks / [time between first and last lick]) within 1 Hz of the extra-trial bout (Figure 4). To test correlations between spike rates and licking behavior of the rat, we took all licking bouts with more than one lick. We quantified the licking frequency between the first and last lick of the bout as well as the spike frequency for each unit. We then performed Pearson’s correlations between the spike rate and licking frequency for each unit. Finally, we split the (positively, negatively and unmodulated) populations in a licking-frequency correlated group and an uncorrelated group and made licking-bout triggered PETHs for all six groups (Supplementary Figure S2).

### Statistical Analysis

A bootstrapping method was used to determine whether spiking activity of individual units was modulated by a specific segment of correct behavioral performance. First, we aligned the spiking activity of an individual unit to the Hit time stamps (first lick within decision window of a “Go” trial) and computed the Δ spike rate (mean spike rate in the 1 s windows before Hit outcomes minus mean spike rate during the session) of each unit during the 1 s before these Hit outcomes (mock example in Figure 2A). Subsequently, an equal number of random “Hit” time stamps were generated throughout the recording session and the mean Δ spike rate in the 1 s before these randomized “Hit” time stamps was determined. This procedure was repeated 1,000 times after which a distribution of Δ spike rate to the randomized triggers was constructed and compared to the Δ spike rate upon true Hit time stamps. If the recorded Δ spike rate of a unit was in the 1st percentile of its (randomized) bootstrap distribution it was grouped in the negatively modulated spike rate group (reduced spiking activity in 1 s window before Hit outcomes) and conversely, when the value was in the 99th percentile the unit was placed in the positively modulated spike rate group (increased spiking activity in 1 s window before Hit outcomes; Figures 2C,D). Units that did not fall in either group were considered unmodulated.

We performed Kolmogorov-Smirnov tests to check whether our data was normally distributed. To determine correlations between spike rates and Δ spike rate and between licking probability and trial number (Figures 1E, 2E), Pearson’s correlation (α < 0.05) was used. To compare medians of two populations, Wilcoxon signed-rank tests were used (α < 0.05). Finally, to compare three or more groups, Kruskal-Wallis tests were used followed by *post hoc* multiple comparison tests. In case of multiple testing, a Bonferroni correction was used.

## Results

To answer how mPFC neurons encode tactile decision making, motivated behavior and learning from mistakes, we trained rats in a head-fixed whisker-based object localization task. Data of three rats reached selection criteria for quality of electrophysiological recordings and behavioral performance. In this task, rats learned to report proximal (“Go”) location of an object by licking for a water reward (20–40 μl) while refraining from licking when the object was placed distally (“No-go”) to avoid a TO punishment (Figures 1A,B). “Go” trials were called Hit if the rat licks and Miss when the rat does not, conversely, licking during a “No-go” trial resulted in a FA, while refraining from licking resulted in a CR. Rats completed on average 147 trials per session (range 77–253 trials). After the object moved into the whisker field, rats started whisking extensively and many touches followed, both during “Go” and “No-go” trials (Supplementary Figure S1). Rats readily distinguished between “Go” and “No-go” positions and licked preferentially during “Go” trials over “No-go” trials (Proportion correct: “Go” 0.67 ± 0.13; “No-go” 0.82 ± 0.10; All 0.74 ± 0.07, mean ± standard deviation; Figures 1C–E, Table 1). Catch trials were intermixed to test whether rats relied on whisker information to determine task outcome. Rats typically did not lick during Catch trials (Proportion not licked: 0.98 ± 0.04, mean ± standard deviation; Figures 1C–E). Overall performance was stable between sessions, although rats tended to show a higher probability to lick during early trials compared to late trials (“Go”: *R* = −0.86, *P* = 1.8*10^−5^; “No-go”: *R* = −0.80, *P* = 1.8*10^−4^; Figure 1E, *n* = 16 sessions in *N* = 3 rats), which could reflect satiety from earned rewards.

**Table 1:** Complete overview of behavioral and spiking parameters.

Accuracy behavioral performance (proportion correct, Figure 1D)				
Trial type	Go	No-go	Catch	All
	0.67 ± 0.13	0.82 ± 0.10	0.98 ± 0.04	0.74 ± 0.07
Baseline spiking properties in Hz (Figure2F)				
Population	Neg. modulated	Unmodulated	Pos. modulated	
	**2.48**, 1.36/4.94	**0.77**, 0.11/1.98	**2.06**, 0.99/4.5	
Neural representation of motor output and context in Hz (Figure 3E3)				
Population	Neg. modulated	Unmodulated	Pos. modulated	
No licks	**2.49**, 1.27/4.98	**0.80**, 0.07/2.06	**2.09**, 0.83/4.50	
Extra-trial licks	**0.84**, 0.08/1.80	**0.26**, 0/1.49	**2.19**, 0.87/4.64	
Intra-trial licks	**1.26**, 0.25/3.19	**0.60**, 0.04/1.91	**4.26**, 1.80/8.65	
Neural representation of context for matched behavior in Hz (Figures 4C–E)				
Population	Neg. modulated	Unmodulated	Pos. modulated	
Extra-trial licks	**0.85**, 0/3.20	**0.34**, 0/2.56	**1.93**, 0.17/6.18	
Intra-trial licks	**0.79**, 0/2.77	**0.19**, 0/1.71	**4.70**, 1.08/9.26	
Performance as function of previous trial (proportion licked, Figures 5A1,A2)				
	Post-Hit	Post-Miss	Post-FA	Post-CR
No-go	**0.20**, 0.11/0.29	**0.12**, 0/0.25	**0**, 0/0.23	**0.10**, 0.08/0.17
Go	**0.72**, 0.57/0.86	**0.56**, 0.39/0.72	**0.80**, 0.67/1.00	**0.67**, 0.53/0.75
Spike rate change upon feedback (∆ spike rate in Hz, Figures 5B,C)				
	Neg. modulated		Pos. modulated	
Hit_CR_	**−0.65**, −1.61/0.05		**1.06**, 0.02/5.10	
Hit_FA_	**−0.64**, −1.47/0.01		**0.25**, −0.51/1.60	
FA_CR_	**−0.08**, −0.59/0.36		**0.07**, −0.16/1.39	
FA_FA_	**−0.60**, −1.74/−0.20		**0.06**, −0.60/2.1	
Accuracy behavioral performance under dynamic conditions (proportion correct, Figure 6B)				
Trial type	Go	Hard	Normal	Easy
	0.62 ± 0.04	0.31 ± 0.04	0.18 ± 0.03	0.07 ± 0.02
Neuronal representation of dynamic conditions (∆ spike rate in Hz, Figures 6C,D)				
	Neg. modulated		Pos. modulated	
Hit	**−0.44**, −0.70/−0.02		**1.61**, 0.49/2.36	
Hard FA	**−0.07**, −0.80/0		**1.45**, 0.32/2.72	
Normal FA	**−0.04**, −0.98/0		**0.83**, 0.16/2.18	
Easy FA	**−0.02**, −1.35/−0.05		**−0.09**, −0.78/1.12	

*Values are in average ± stdev (Figures 1D, 6B) or **median**, 1st – 3rd quartile range*.

To monitor mPFC neuron spiking activity, 64-channel silicon probes were implanted. Spikes were analyzed and clustered off-line (Figures 1F1,F2), which resulted in 204 well-isolated units distributed over the dorsal-ventral axis and throughout mPFC layers. FSUs (peak-to-trough time <0.5 ms and half-peak time <0.25 ms, gray), were excluded from subsequent analyses (Figures 1G1,G2). RSUs represent putative excitatory pyramidal neurons for which we found strong correlations between specific trial parameters and spike rates in a subset of units (example in Figures 1H1–H3). Thus, spiking in a subpopulation of mPFC RSU units strongly correlated with behavioral performance.

### Unit Classification

To identify which units were significantly modulated during correct performance of the task, we performed a bootstrap analysis on the spike train of each unit (Figures 2A–C). We found positively and negatively modulated units (Figures 2B–D, magenta, *n* = 31 and cyan, *n* = 48 respectively) as well as unmodulated units (gray, *n* = 112). The modulated populations both had higher median spike rates compared to unmodulated units (Spike rate values: median, 1st/3rd quartile: positively modulated 2.06, 0.99/4.5 Hz; unmodulated 0.77, 0.11/1.98 Hz; negatively modulated 2.48, 1.36/4.94 Hz, *p* = 2*10^−8^, Kruskal-Wallis test; Figure 2F) and there was a strong correlation of larger Δ spike rate for higher mean spike rates (Figure 2E, positively modulated *R* = 0.62, *p* = 2*10^−4^; negatively modulated *R* = −0.77, *p* = 1.3*10^−10^, Pearson’s correlation).

### mPFC Units Encode Motor Output and Context

The bootstrap analysis identified units with spike rates that were positively modulated (Figure 3A), unmodulated (Figure 3B) or negatively modulated (Figure 3C) during correct “Go” trials. Units with positively modulated spiking activity (*n* = 31) were clearly excited before Hit outcomes and remained excited during reward delivery and consumption (blue, Figure 3A1). Similarly, during FA trials the average spike rate of positively modulated units increased before FA outcomes. As soon as rats received feedback on trial outcome however (i.e., learned that it made a mistake), spike rates returned to baseline (red, Figure 3A2). During Miss and CR trials, spike rates of the positively modulated population remained stable (purple and orange respectively, Figures 3A1,2). Unmodulated units (*n* = 112) did not show spike rate changes for any trial type (Figure 3B). The negatively modulated population (*n* = 48) showed a clear reduction in spike rate before, during and after Hit (Figure 3C1) and FA outcomes (Figure 3C2). Similar to the positively modulated units, spike rates of the negatively modulated population returned to baseline quickly after outcome of FA trials. Thus, neurophysiological correlates during “No-go” trials closely resembled those during “Go” trials, up to the moment that sensory feedback signaled whether behavioral output was appropriate.

Both the positively and negatively modulated populations showed obvious spike rate changes during trials in which rats licked, independent of reward delivery (Hit and FA) and spike rate changes were absent from non-lick trials (Miss and CR). This may suggest that spike rate modulations during trials in which rats were licking represented motor output. To further quantify correlations between modulation of mPFC spiking activity and licking behavior, we analyzed licking behavior for each trial type (Figure 3D) and found that the time course of licking matched changes in spike rate. Most licks occurred during Hit and FA trials, but rats occasionally licked outside trials. These extra-trial licks presumably represent licking behavior without the expectation to earn water rewards (see e.g., the ticks at 0.5–1.5 s for CR and Miss trials in Figure 1C1). It is thus tempting to assume that these licks were not as strongly driven by expectance to receive rewards compared to intra-trial licks and we therefore used this distinction as a control for motor behavior. Thus, to test whether Δ spike rates were related to motor output, we analyzed PETHs of spike rates centered around the start of lick bouts for both intra-trial bouts and extra-trial bouts (Figures 3A3–C3). Both positively and negatively modulated populations had stronger spike rate modulation around lick start for intra-trial licks compared to extra-trial licks (compare amplitude of solid and dashed lines for intra- and extra-trial licking respectively, Figures 3A3–C3). We additionally quantified the contribution of motor output (i.e., licking) to spike rate modulation by comparing spike rates during no licks vs. extra-trial licks. We found that spike rates of the unmodulated and negatively modulated population during extra-trial licks significantly decreased relative to no-lick episodes (neg. modulated *p* = 5*10^−7^; unmodulated *p* = 1*10^−4^; Wilcoxon signed-rank test with Bonferroni correction; Figure 3E3). Spike rate modulation was not observed in the positively modulated population upon comparison of no-lick and extra-trial licking episodes, indicating that spiking in this population is insensitive to motor output (pos. modulated *p* > 0.05).

Since intra-trial licking is associated with reward expectation, intra-trial licks probably represent a combination of motor output and contextual value (i.e., reward delivery). We thus quantified spike rates between intra- and extra-trial licking to determine whether context (i.e., reward delivery) impacted spike rate beyond motor output only (extra-trial licks). All three populations showed spike rate modulation with respect to the cognitive context and have a higher median spike rate during intra-trial compared to extra-trial licking (pos. modulated *p* = 0.01; unmodulated *p* = 0.03; neg. modulated *p* = 0.02; Wilcoxon signed-rank test with Bonferroni correction; Figure 3E3). This indicates that context increased spike rates consistently in all three populations.

Since we cannot rule out that fine-scale differences in behavior during intra- and extra-trial licks may underlie differences in spike rate modulation, we quantified licking behavior after intra- and extra-trial licking bout starts (Figure 3D3). Indeed, intra-trial licking frequencies were higher compared to extra-trial licking frequencies. When different licking frequencies underlie spike rate differences between intra- and extra-trial lick bouts, spike rates should correlate with licking frequency. For a subset of units, spike rate indeed significantly correlated to licking rate (Supplementary Figures S2A1,A2). Moreover, differences in spike rates between intra- and extra-trial lick bouts persisted when licking frequency-correlated units were excluded from the analysis (Supplementary Figures S2B2,D2).

### Positively Modulated Units Represent Contextual Value

Finally, we matched individual extra-trial lick bouts with intra-trial lick bouts based on similar behavioral statistics (i.e., lick frequency, number of licks and bout duration) to rule out behavioral differences and isolate contextual value. We found that spike rate modulation during licking was comparable between intra-trial and extra-trial licking bouts when behavioral statistics were matched for the negatively and unmodulated populations (*p* > 0.05, Wilcoxon’s signed-rank test with Bonferroni correction, Figure 4), which indicates that spike rate modulation during licking is due to motor output and not contextual value. For positively modulated units, spike rates during intra-trial licking bouts continued to be significantly higher compared to extra-trial licking bouts after matching behavioral characteristics (Figure 4E), indicating that context but not motor output impacted spike rates in this population (pos. modulated *p* = 0.01, Wilcoxon’s signed-rank test with Bonferroni correction).

We also changed the cognitive context by doubling the water reward or by delaying reward or punishment but these experimental manipulations did not consistently change the behavior or spike rates and these manipulations were therefore not used for further in-depth analyses (Supplementary Figure S3). Collectively, our data suggest that motor behavior and cognitive context contribute to spike rate modulation during task execution, but modulation depth depends on the neuronal population involved.

A subset of “Go” trials is associated by the absence of licking, leading to “Miss” categorization. As a consequence, CR trials may be the outcome of a “No-go” miss instead of an active decision to identify the trial as “No-go.” To test whether withholding licks during “No-go” trials is a passive process or is actively encoded by the mPFC, the bootstrap analysis was applied again (Figures 2A–C) to classify units based on Δ spike rate during correct “No-go” performance. We found positively (*n* = 9), negatively (*n* = 12) and unmodulated units (*n* = 170; 12 out of 21 units were also modulated during Hit trials; Supplementary Figure S4). We quantified the median spike rate of the “No-go” CR-negatively modulated population during the 1 s before “Go”-Hit outcomes and found that it was not negatively modulated (data not shown). In contrast, the “No-go” CR-positively modulated population showed a reduction in spike rate in the 1 s window before Hit outcomes (*p* = 0.006 sign test with Bonferroni correction; Supplementary Figure S4A3). Thus, the presence of CR-modulated units supports the hypothesis that “No-go” trials are actively encoded in mPFC and rats actively determine the outcome of both “Go” and “No-go” trials.

### Negatively Modulated Units Signal Updating of Task Rules on a Trial-to-Trial Basis

The mPFC is involved in monitoring and updating behavior in highly dynamic environments. During the behavioral task, rats constantly received feedback on performance through water rewards for Hit trials and sound and TOs for FA trials. The general assumption is that these cues are integrated with existing internal rule representations and change behavioral strategies to drive rewarded behavior (Dalley et al., 2004; Rich and Shapiro, 2009; Durstewitz et al., 2010; Horst and Laubach, 2012; Narayanan et al., 2013). In accordance, rats were more likely to lick in “No-go” trials following a Hit trial compared to “No-go” trials following a FA trial (*p* = 0.012, Wilcoxon signed-rank test with multiple comparisons, significant difference between “No-go” trials after Hit or FA outcome; Proportion licked: Hit 0.20, 0.11/0.29; FA 0, 0.0/0.23, values represent median, 1st/3rd quartile; Figure 5A1). This effect was not observed for “Go” trials (Figure 5A2), since performance during ongoing trials did not depend on the outcome of the previous trial. Thus, rats adapt behavior on a trial-to-trial basis to avoid consecutive FA trials and associated TO punishments.

To address the question whether mPFC spiking correlates with the trial-to-trial behavioral shifting, we tested correlations between mPFC spiking activity during trial outcome (feedback) of Hit and FA trials and subsequent trial performance. Thus, we took the PETHs of the modulated units for Hit trials followed by a “No-go” trial (Hit_CR_ and Hit_FA_) and similarly for FA trials followed by a “No-go” trial (FA_CR_ and FA_FA_; Figures 5B,C). We selected units that were recorded in sessions that contained both Hit_CR_ and Hit_FA_ or both FA_CR_ and FA_FA_ trials for the following analyses. We computed the average spike frequency of units in the time window where feedback was received and integrated (i.e., 0.2–3 s after outcome). We hypothesized differential spiking activity when the behavior on the next trial corresponded with feedback signals in the previous trial (i.e., keep licking after reward: Hit_FA_; or stop licking after punishment: FA_CR_) compared to no correspondence (Hit_CR_ and FA_FA_). Neither positively modulated units, nor negatively modulated units showed different spike rates between Hit_CR_ and Hit_FA_ (Δ spike rate: pos. modulated Hit_CR_ 1.06, 0.02/5.10 Hz; Hit_FA_ 0.25, −0.51/1.59 Hz; neg. modulated Hit_CR_ −0.65, −1.61/0.05 Hz; Hit_FA_ −0.64, −1.47/0.01, values are median, 1st/3rd quartile; *p* > 0.05, Wilcoxon signed-rank test with Bonferroni correction; Figures 5B2,3). Thus, spike rates during correct “Go” trials did not predict performance during subsequent “No-go” trials. Similarly, positively modulated units showed comparable spike rates during feedback of FA_CR_ and FA_FA_ trials (FA_CR_ 0.07, −0.16/1.39 Hz; FA_FA_ 0.06, −0.60/2.12 Hz, values are median, 1st/3rd quartile; *p* > 0.05, Wilcoxon signed-rank test with Bonferroni correction; Figure 5C2). Surprisingly, negatively modulated units had lower spike rates during FA_FA_ trial feedback (when the behavior was not changed) compared to FA_CR_ trials, when rats updated their behavior and correctly performed during the next “No-go” trial (FA_CR_ −0.08, −0.59/0.36 Hz; FA_FA_ −0.60, −1.74/−0.20 Hz, values are median, 1st/3rd quartile; *p* = 0.012, Wilcoxon signed-rank test with Bonferroni correction; Figure 5C3). To test whether this effect was robust, the two most active neurons were removed from the population, but this did not affect statistical outcome (*p* = 0.043, Wilcoxon signed-rank test with Bonferroni correction).

Additional evidence for mPFC encoding of behavioral shifting can be found in spike rates of the CR-modulated units. The positive CR-modulated units did not encode behavioral updating after error feedback (*n* = 5; Supplementary Figures S5A,B), but the negative CR-modulated population spiked more during feedback integration of FA_FA_ trials compared to FA_CR_ trials (*p* = 0.03, Wilcoxon signed-rank test with Bonferroni correction, *n* = 8; Supplementary Figure S5C). We therefore propose that both negative Hit-modulated and negative CR-modulated populations may carry the neurophysiological representation of adaptive behavior in response to unsuccessful trial outcome and in turn may drive trial-to-trial learning.

### Spike Rate Increase in Positively Modulated Units Could Predict Inhibitory Control

It has been suggested that mPFC recruitment depends on the level of task difficulty (Euston et al., 2012; Zhang et al., 2014; Kamigaki and Dan, 2017) and that increasingly difficult tasks involve mPFC more strongly (Chudasama and Muir, 2001; White et al., 2018; Wu et al., 2018), while others suggest that mPFC encodes certainty (Hanks et al., 2015). Additionally, correct mPFC spiking activity may be essential for impulse control (Narayanan and Laubach, 2006, 2009; Chudasama et al., 2012; Gourley and Taylor, 2016). Therefore, in one session each day, we randomly intermixed two extra “No-go” locations, in addition to the Normal “No-go” location. These locations were chosen such that the Easy location was twice as far from the “Go” location (8 mm) as the Normal location (4 mm) and the Hard location was half as far (2 mm; Figure 6A). The rats performed as expected; they licked mostly for “Go” trials, made a relatively high number of (FA) mistakes for the Hard “No-go” location and progressively fewer mistakes for the Normal and Easy “No-go” locations (Proportion licked “Go”: 62.1 ± 3.8%; Hard “No-go”: 31.4 ± 3.8%; Normal “No-go”: 17.8 ± 3.4%; Easy “No-go”: 6.8 ± 1.7%; mean ± SEM; Figure 6B). During these sessions both the positively and negatively modulated populations (*n* = 20 and *n* = 35 respectively) responded similarly relative to regular sessions (compare Figure 3C with Figures 6C1,D1) with prolonged increase/decrease of spike rates during licking and reward consumption in Hit trials and rapid return to baseline spiking after Normal FAs (Figure 6C1). “No-go” trial type did not determine the spike rate of the negatively modulated units during FA and CR trials (Figure 6C, CR not shown). Spiking of the positively modulated units was also not dependent on the type of CR trials (data not shown). However, spike rates of positively modulated units differed between distinct types of lick trials (Δ spike rate: Hit 1.61, 0.49/2.36 Hz; Hard “No-go” 1.45, 0.32/2.72 Hz; Normal “No-go” 0.83, 0.16/2.18 Hz; Easy “No-go” −0.09, −0.78/1.12 Hz, values are median, 1st/3rd quartile; *p* = 0.01, Kruskal-Wallis test; Figure 6E). More specifically, positively modulated units exhibited reduced spiking in the 0–2 s prior to outcomes of Easy FA compared to the same time-window during Hard FA trials (*p* = 0.004, Wilcoxon signed-rank test with Bonferroni correction; Figure 6D3) and during Normal FA trials (*p* = 0.035, Wilcoxon signed-rank test with Bonferroni correction). Thus, mPFC spiking activity scales with task difficulty during error trials. Since rats may deploy different cognitive or behavioral strategies to solve Easy vs. Hard “No-go” trials (# of licks *p* = 0.055 and licking peak latency *p* = 0.063; Wilcoxon signed-rank test; Supplementary Figure S6), future work should elucidate which factors underlie the observed correlation between task difficulty and mPFC spiking activity.

## Discussion

The mPFC orchestrates a variety of cognitive functions, such as strategy optimization, reward seeking and inhibitory control (Narayanan and Laubach, 2006; Euston et al., 2012; Narayanan et al., 2013; Orsini et al., 2015; Pinto and Dan, 2015; Kim H. et al., 2016; Luchicchi et al., 2016; Guise and Shapiro, 2017; Kamigaki and Dan, 2017; Malagon-Vina et al., 2018). Pharmacological disturbance of mPFC leads to deficits in behavioral switching (Guise and Shapiro, 2017), stimulus discrimination (Pinto and Dan, 2015) and memory-guided decision making (Lagler et al., 2016), while optogenetic manipulation of mPFC neurons disturbs correct performance of attentional (Wimmer et al., 2015; Kim D. et al., 2016; Kim H. et al., 2016; Luchicchi et al., 2016; Schmitt et al., 2017), working memory (Horst and Laubach, 2012; Kim D. et al., 2016; Bolkan et al., 2017) and stimulus discrimination tasks (Kamigaki and Dan, 2017). Conversely, selectively increasing the activity of mPFC parvalbumin (PV) or vasoactive intestinal peptide (VIP) expressing neurons can increase performance in an attention task and a stimulus discrimination task respectively (Kim H. et al., 2016; Kamigaki and Dan, 2017). Neurophysiological correlates of both somatosensory stimulation and decision making were uncovered in the mPFC and the mPFC is necessary for passive tactile decision making (Le Merre et al., 2018). However, mPFC involvement in active tactile decision making and short-timescale behavioral updating has remained understudied. Here, we report mPFC neurophysiological representations of decision making during a “Go”/“No-go” whisker-based, object localization task (O’Connor et al., 2010a; Pammer et al., 2013).

We performed extracellular electrophysiological recordings in the mPFC of rats in combination with rigorous cluster analysis to reach single-unit resolution (Csicsvari et al., 2003; Barthó et al., 2004; Schmitzer-Torbert et al., 2005; Rossant et al., 2016) and aligned spiking responses to specific epochs of task performance. We found units showing positive spike rate modulation (increased spiking), negative modulation (decreased spiking) or no modulation (no change) when aligned to correct “Go” performance, which is comparable to sensory, motor, associative and prefrontal cortices (Narayanan and Laubach, 2009; Totah et al., 2009; O’Connor et al., 2010b, 2013; Hanks et al., 2015; Zagha et al., 2015; Kim H. et al., 2016; Ebbesen et al., 2017; Le Merre et al., 2018). We assumed that populations with specific modulation characteristics represent segregated (and potentially competing) microcircuits (Halladay and Blair, 2015; Kim H. et al., 2016) and thus analyzed these populations separately in subsequent in-depth analyses. We found that: (1) modulation of spike rates coincides with decision making and motor output and modulation depth correlates to cognitive context; (2) low excitation of positively modulated units during Easy “No-go” trials predicts FA mistakes; and (3) units negatively modulated during correct “Go” behavior encode behavioral state updating by increased spiking after an error was made.

### mPFC as a Single Functional Area

Even though there is evidence showing differential functional roles and innervation patterns of distinct mPFC areas (Heidbreder and Groenewegen, 2003; Euston et al., 2012; Gourley and Taylor, 2016), we chose not to subdivide the mPFC. We found significantly modulated units over the entire dorso-ventral extent of the mPFC without different proportions of significantly modulated units along the dorso-ventral axis (Supplementary Figure S7). Classical cytoarchitectonic divisions of mPFC (i.e., AC, PL and IL cortex) or divisions into the dmPFC and the vmPFC (Gabbott et al., 2005; Luchicchi et al., 2016) lead to ambiguous and potentially artificial subdivisions of the mPFC (Heidbreder and Groenewegen, 2003; Euston et al., 2012; Hardung et al., 2017). The absence of a dorso-ventral distinction of unit task-selectivity in our dataset strengthens our confidence that for our task mPFC subdivision is unnecessary and potentially counterproductive.

### mPFC Processing of Tactile Behavior

Primary and/or secondary sensory cortices govern stimulus detection after low intensity sensory stimuli (Yang et al., 2016; Le Merre et al., 2018), whereas CA1 and mPFC are necessary for correct performance in detection tasks (Pinto and Dan, 2015; Le Merre et al., 2018). However, it remains unknown how sensory information is integrated into the mPFC during stimulus discrimination and how the mPFC influences sensory processing during tasks that involve sensation. Activity of mPFC axons increases stimulus detection and discrimination by top-down center-surround contrast enhancement in V1 (Zhang et al., 2014) and are necessary to respond to low-contrast stimuli during conditioning (Wu et al., 2018). The mPFC also increases stimulus detection by driving modality-dependent attention through the thalamus (Wimmer et al., 2015). The mPFC does not receive strong direct inputs from S1 (Hoover and Vertes, 2007; DeNardo et al., 2015) or somatosensory thalamic nuclei VPM and PoM (Hoover and Vertes, 2007), although somatosensory field potentials are observed in the mPFC after whisker stimulation in both anesthetized rats (Martin-Cortecero and Nuñez, 2016), awake untrained mice and trained mice (Le Merre et al., 2018). We did not observe robust changes in spiking upon touch in the mPFC (data not shown). Nonetheless, tactile information probably should be present in the mPFC to correctly drive tactile decision making. We did see a fast increase in local field potential amplitude (latency of approximately 50 ms, data not shown) after the first touch for each trial, but we cannot exclude the possibility that this local field potential carries multimodal information. When the mPFC does not encode sensory information directly, the alternative explanation could be that sensory information from primary sensory cortices is summarized by feature extraction and/or categorization (Hanks et al., 2015) in an upstream brain region, e.g., the more dorsal parts of frontal cortex (Sreenivasan et al., 2017) or the orbitofrontal cortex as has been suggested by Euston et al. (2012), since there is strong innervation from both these regions to mPFC (Hoover and Vertes, 2007). During our task we do not identify specific sensory spiking patterns. However, it remains enigmatic whether this is due to absence of sensory signals altogether or because the sensory information is packaged in a way that we cannot yet recognize and decode in separation from the motor and cognitive components of the task.

### Recorded Populations and Their Characteristics

In our dataset, we aimed to address coding properties of pyramidal projecting neurons and therefore excluded putative GABAergic interneurons based on their fast-spiking waveform (Barthó et al., 2004). A small population of interneurons with a regular-spiking waveform may nevertheless remain in our dataset (Kim D. et al., 2016). In addition, histological recovery of recording location indicated that the predominant fraction of units was recorded in the deep layers (layer 5 and 6). Interpretations of the output of the mPFC circuitry may thus be influenced by overrepresentation of units from these layers. This is important to consider since encoding of task-relevant information will almost certainly be layer- and cell-type specific and thus correlate to projection target (Gabbott et al., 2005; Dembrow et al., 2010; Pinto and Dan, 2015; Kamigaki and Dan, 2017). For example, layer 5 units can project to the lateral hypothalamus and carry appetite-related signals or send motor signals to striatum, ventral tegmental area and spinal cord, while units from layer 6 can project to the mediodorsal (MD) thalamus (Vertes, 2002, 2004; Gabbott et al., 2005; Morishima et al., 2011). Additionally, the spiking activity of layer 2 and layer 3 output projections, most notably to basolateral amygdala (Gabbott et al., 2005), need to be determined to generate a comprehensive view on cell-type and layer-specific activity in mPFC during the whisker-guided objection localization task. We have not been able to link our three populations of modulated/unmodulated units to projection target or specific morphological types (Dembrow et al., 2010; Morishima et al., 2011; van Aerde and Feldmeyer, 2015; Kawaguchi, 2017). However, this may be essential for future experiments to increase the interpretability of links between the morphological structure, electrophysiological function, and influence on the behavior of the recorded populations.

### Activity in mPFC Pyramidal Neurons Is More Than Motor Representation

In our study, we used the first lick during the decision window as a discrete trigger to signal decision making, but this means that it is not straightforward to disentangle spiking involved in decision making (e.g., motivation, cognitive context and sensory inputs) from spiking involved in motor output (Horst and Laubach, 2013; Amarante et al., 2017). This could be solved by temporally separating the response moment from motor output (delayed reward), but this goes at the expense of identification of the exact decision moment. Instead, we compared licks during and outside trials to quantify the impact of context on spike rate and “no licks” vs. “extra-trial licks” to quantify the influence of motor output. We show that motor output without contextual value (i.e., lick outside trial) leads to a decrease in spike rates, restricted to the populations that showed either no or negative modulation of spike rate during correct “Go” performance. In contrast, the population that showed positive modulation of spike rate during correct “Go” behavior did not show spike rate modulation by motor output. However, the positively modulated population showed a significant difference in spike rate during licks with and without trial-context (Figures 3E3, 4E), indicating a potential influence of motivation or attention processes on spiking. Collectively, cognitive context and motor output influence spiking in mPFC. Spike rate modulation by motor output and context could ultimately be the outcome of excitability changes by neuromodulatory inputs from e.g., the dopaminergic or cholinergic system (reviewed in Thiele and Bellgrove, 2018) or due to strong interactions with regions involved in appetitive behaviors, such as e.g., the supra-mammillary nucleus and lateral hypothalamus (Ikemoto et al., 2004; Hoover and Vertes, 2007; Reppucci and Petrovich, 2016).

### Correlate of Behavioral Updating

It was proposed previously that mPFC may be critical for task learning, but perhaps not as much during task execution (Liu et al., 2014). This hypothesis is at odds with our finding of trial-to-trial changes in spike rates in the mPFC correlating to task performance. In addition, manipulation of the mPFC during a task shows that mPFC is needed to perform many behaviors, from bottom-up stimulus detection (Le Merre et al., 2018) to attention (Kim H. et al., 2016; Luchicchi et al., 2016) and top-down attentional selection (Wimmer et al., 2015; Schmitt et al., 2017). The activity of the mPFC contains short-term memories of actions and their consequences (Narayanan and Laubach, 2008; Horst and Laubach, 2012). Additionally, the mPFC contains a physiological representation of current task rules or strategies to solve the task (Durstewitz et al., 2010; Cho et al., 2015; Malagon-Vina et al., 2018). The mPFC, thus, has access to all information needed to compute strategies to optimize action outcome. Behavioral updating in mPFC is signaled by theta frequency oscillations (Narayanan et al., 2013) and it could be that spiking of the negatively modulated population is phase locked to theta (Horst and Laubach, 2013; Amarante et al., 2017) to broadcast relevant updates to the behavioral state to downstream targets (Fries, 2005; Womelsdorf et al., 2007). Furthermore, the mPFC has indirect control over actions, i.e., optogenetically stimulating efferent projections from the mPFC to the nucleus accumbens core (NAc) and the periventricular nucleus of thalamus (PVT) leads to enhancement or suppression of reward seeking behaviors after negative outcomes (Kim et al., 2017; Otis et al., 2017). Behavioral updating also depends on mPFC to MD thalamus output (Marton et al., 2018) and risky decision making after negative outcomes involves VTA to mPFC projection (Verharen et al., 2018). In the current work, we cannot show causality of spiking rates to changes in behavior. It could be that mPFC spike rate changes during behavioral updating are simply caused by the same upstream neuronal processes, or that it is not the spiking rate change, but rather phase locking of spiking to oscillations that result in behavioral updating (Fries, 2005; Narayanan et al., 2013; Amarante et al., 2017). Furthermore, it remains to be determined whether the functional populations of (positively modulated, unmodulated and negatively modulated) units represent anatomically distinct groups or heterogeneous populations.

### mPFC Excitation Needed to Prevent Avoidable Mistakes

We show that insufficient spiking in positively modulated units predicts FA mistakes when task conditions are easy. We hypothesize that this may be due to either a lack of top-down attentional filtering (Dalley et al., 2004; Zhang et al., 2014; Wimmer et al., 2015; Kim H. et al., 2016; Luchicchi et al., 2016) or due to insufficient inhibitory control (Narayanan and Laubach, 2006, 2009; Chudasama et al., 2012; Luchicchi et al., 2016; Kamigaki and Dan, 2017). The mPFC is an important area for attentional processing and activation of mPFC projections have been shown to increase stimulus detection and contrast in primary visual cortex (V1; Zhang et al., 2014). Similarly, mPFC projections to the pons are selectively necessary to detect low-contrast stimuli during conditioning (Wu et al., 2018). Additionally, mPFC to thalamus projections are needed for proper selection of sensory modality (Wimmer et al., 2015) and top-down input from mPFC to claustrum is needed for correct attention behavior (White et al., 2018). Thus, the mPFC is driving top-down stimulus-response associations. In our experiment, however, we assume that the Easy “No-go” position is easy to distinguish from the “Go” position and that bottom-up input should therefore be sufficient to select the proper action. Thus, we suggest that insufficient excitation of mPFC neurons through malfunctioning of the mPFC-driven inhibitory control mechanisms leads to FA errors. Under Normal and Hard conditions, the Δ spike rate during the low number of errors of impulsivity could get lost in the larger number of errors of discrimination. The link between impulsivity and reduced mPFC spiking is further supported by findings that reduction of mPFC activity leads to more impulsive prematurely expressed responses (Narayanan and Laubach, 2006, 2009; Kim H. et al., 2016; Luchicchi et al., 2016; Hardung et al., 2017) and FAs (Kamigaki and Dan, 2017).

### Final Remarks

In summary, we quantified neurophysiological representations of motivational and cognitive context in the mPFC of rats performing a whisker-based object localization task (Figure 3). We show that a specific fraction of negatively modulated units could underlie behavioral updating on a trial-to-trial basis (Figure 5) and that a subpopulation of mPFC units needs to be sufficiently activated to maintain task performance under dynamic “No-go” task conditions (Figure 6). These results could provide a framework for future studies investigating causality of mPFC neuronal spiking activity to behavioral updating and stimulus detection.

## Author Contributions

RH, HM and CK designed the study. RH performed the experiments. AP, VN and RH designed and programmed the behavioral hardware and software. JL, SB, RH and CK analyzed the data. RH and CK wrote the manuscript with input from all authors.

## Conflict of Interest Statement

The authors declare that the research was conducted in the absence of any commercial or financial relationships that could be construed as a potential conflict of interest.
