# Supplementary figures and images for: Neural Representation of Motor Output, Context and Behavioral Adaptation in Rat Medial Prefrontal Cortex During Learned Behavior
Source: Front Neural Circuits. 2018 Oct 1;12:75. doi: 10.3389/fncir.2018.00075 (PMC6174330; doi:10.3389/fncir.2018.00075)

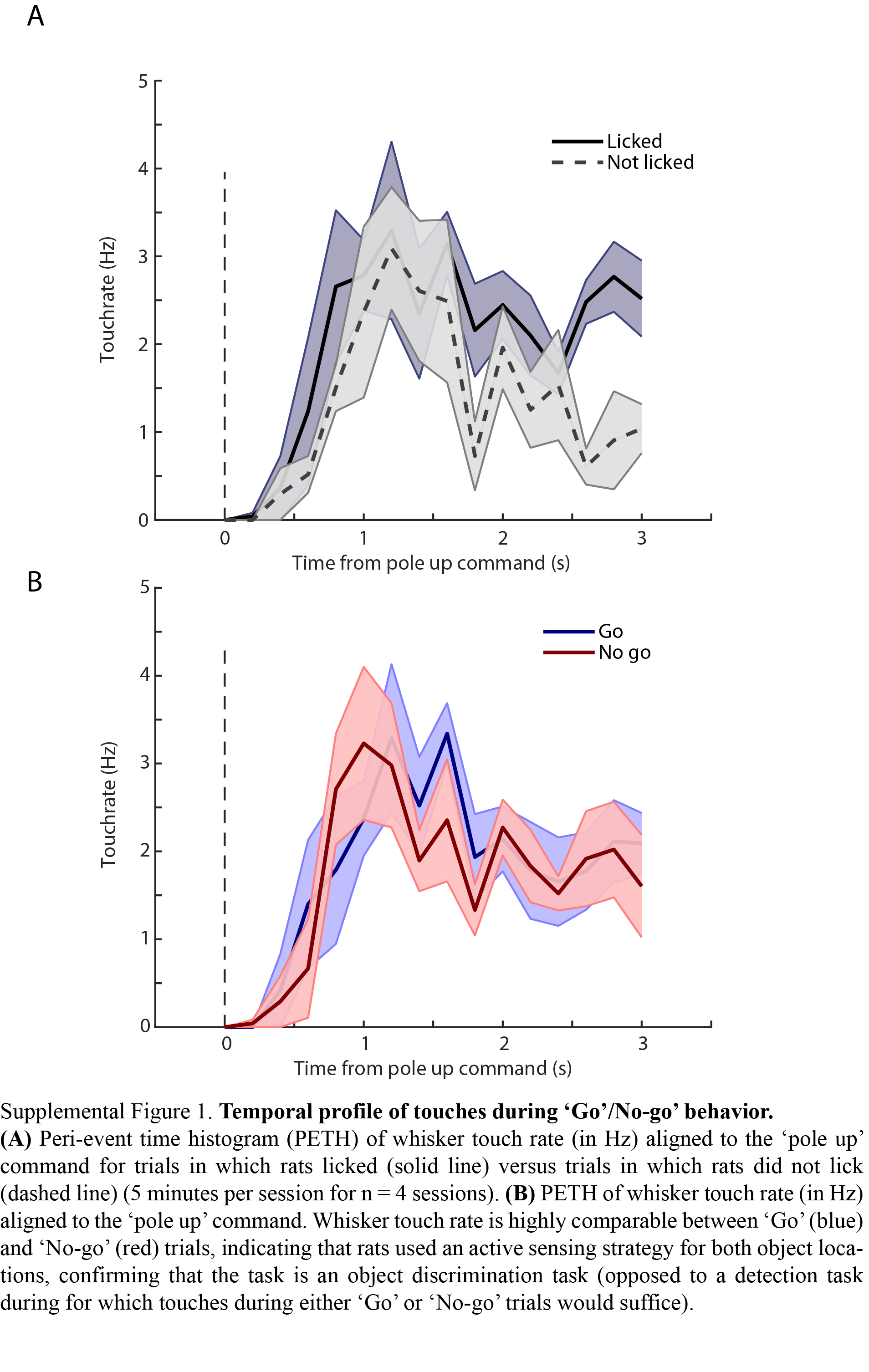

Supplement: Supplementary file 1 [file Image_1.JPEG]

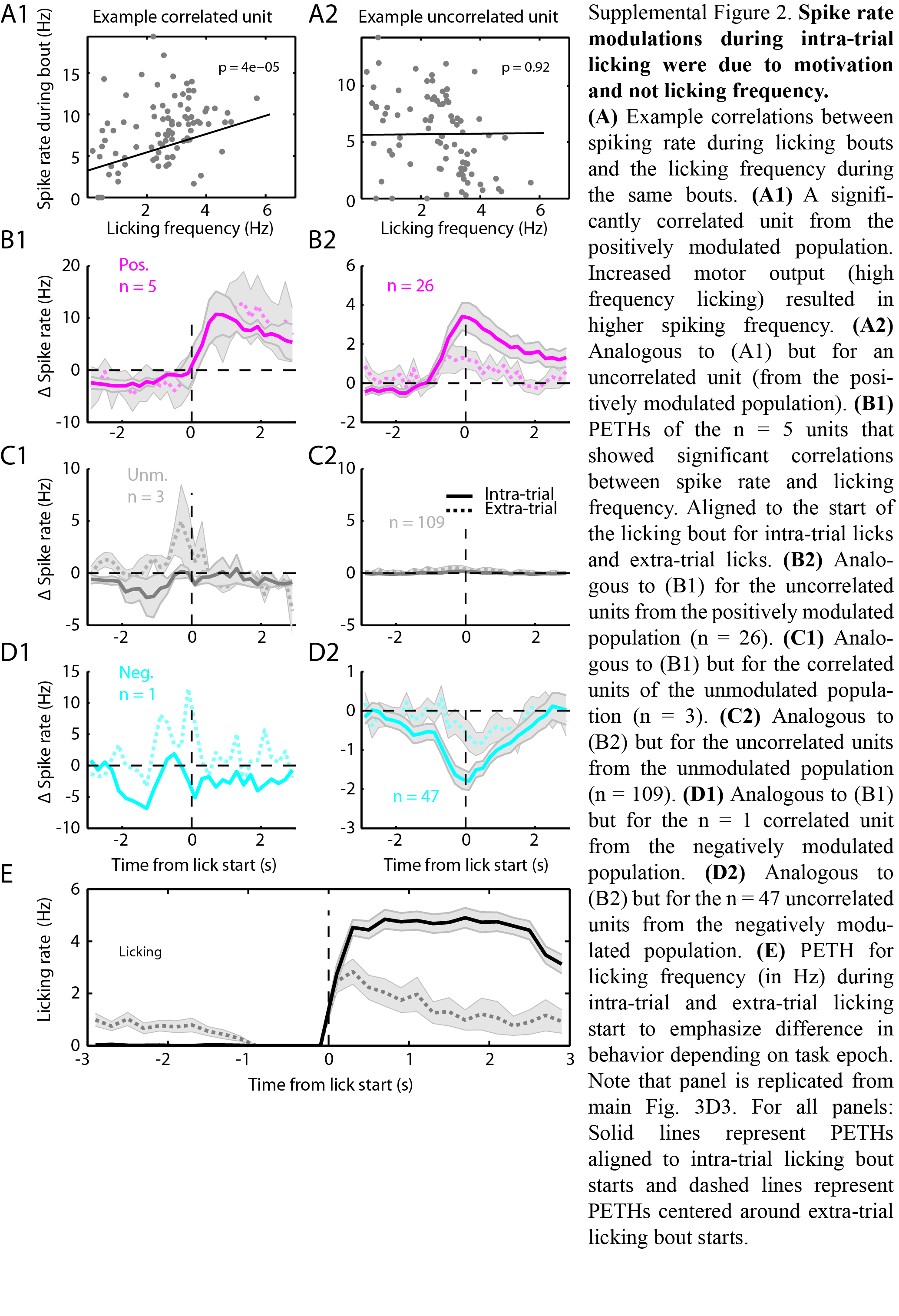

Supplement: Supplementary file 2 [file Image_2.JPEG]

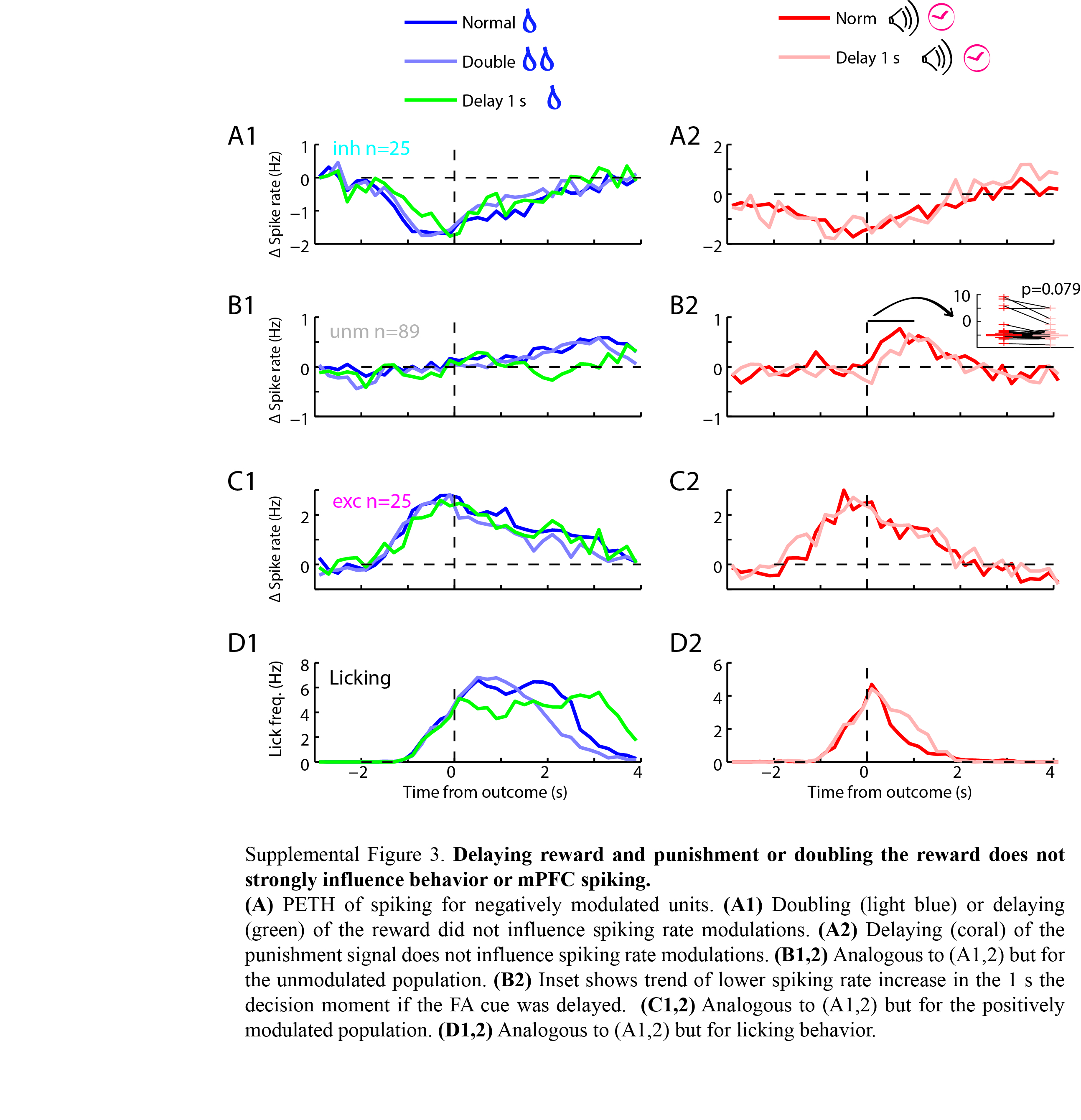

Supplement: Supplementary file 3 [file Image_3.JPEG]

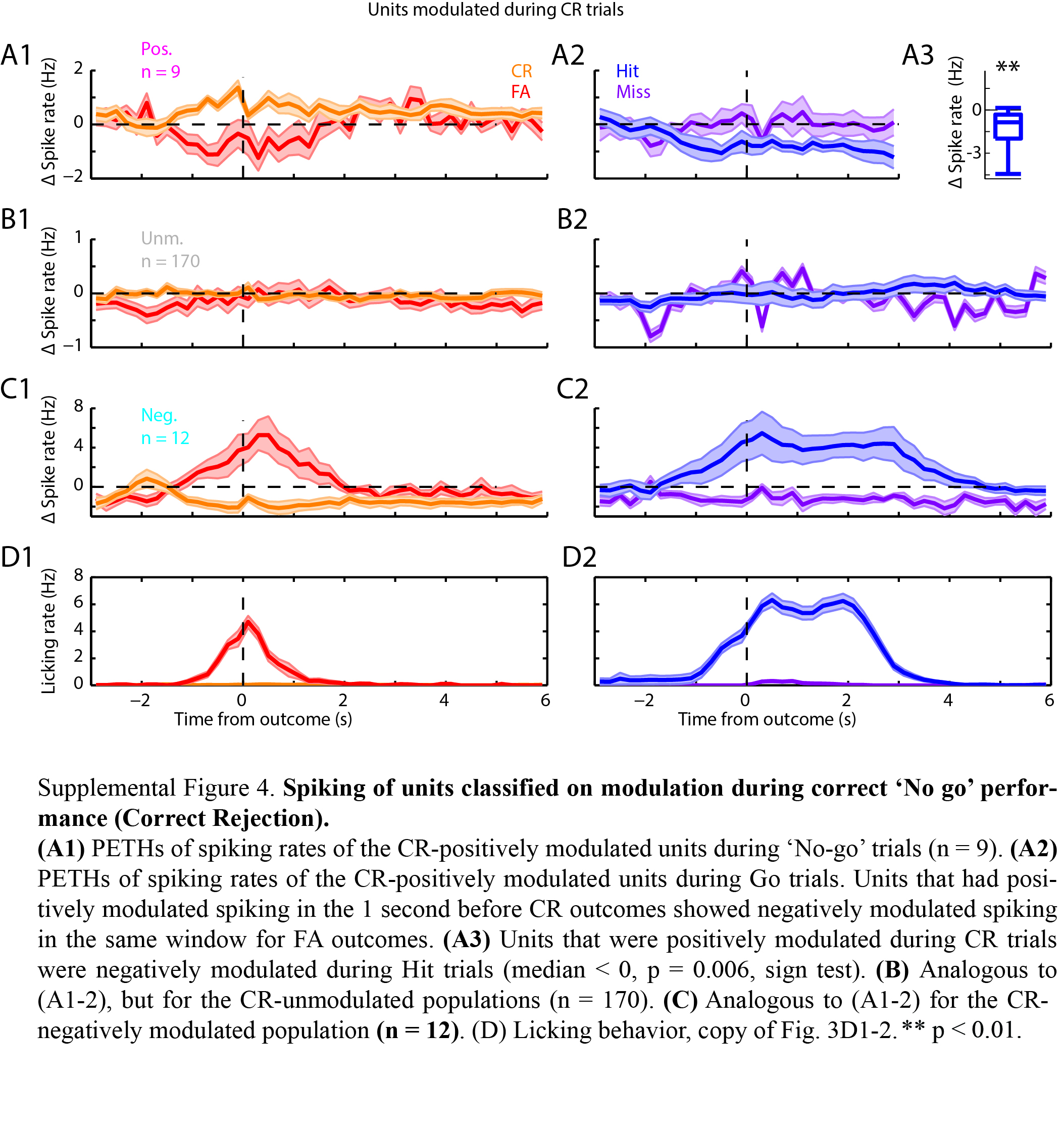

Supplement: Supplementary file 4 [file Image_4.JPEG]

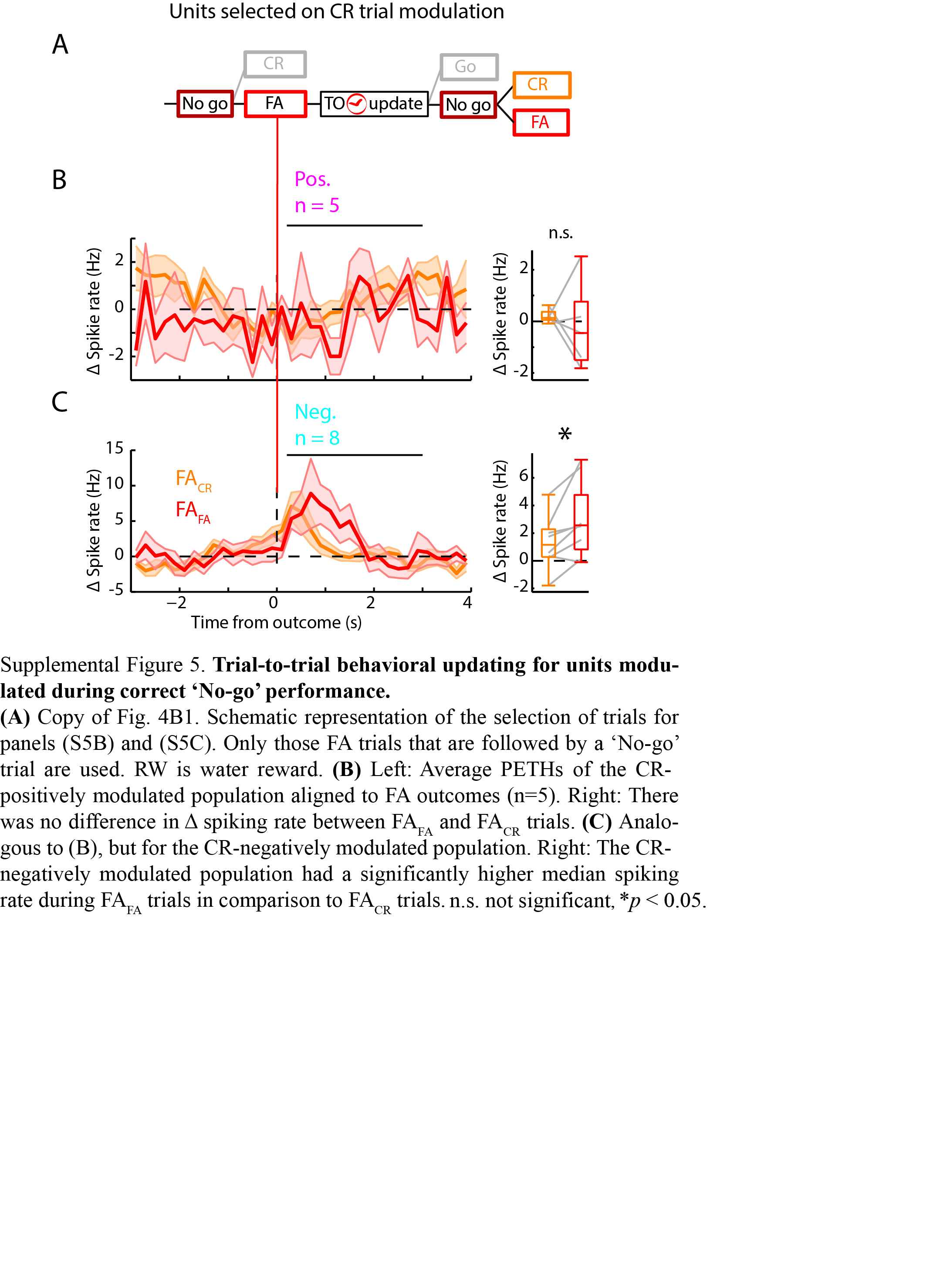

Supplement: Supplementary file 5 [file Image_5.JPEG]

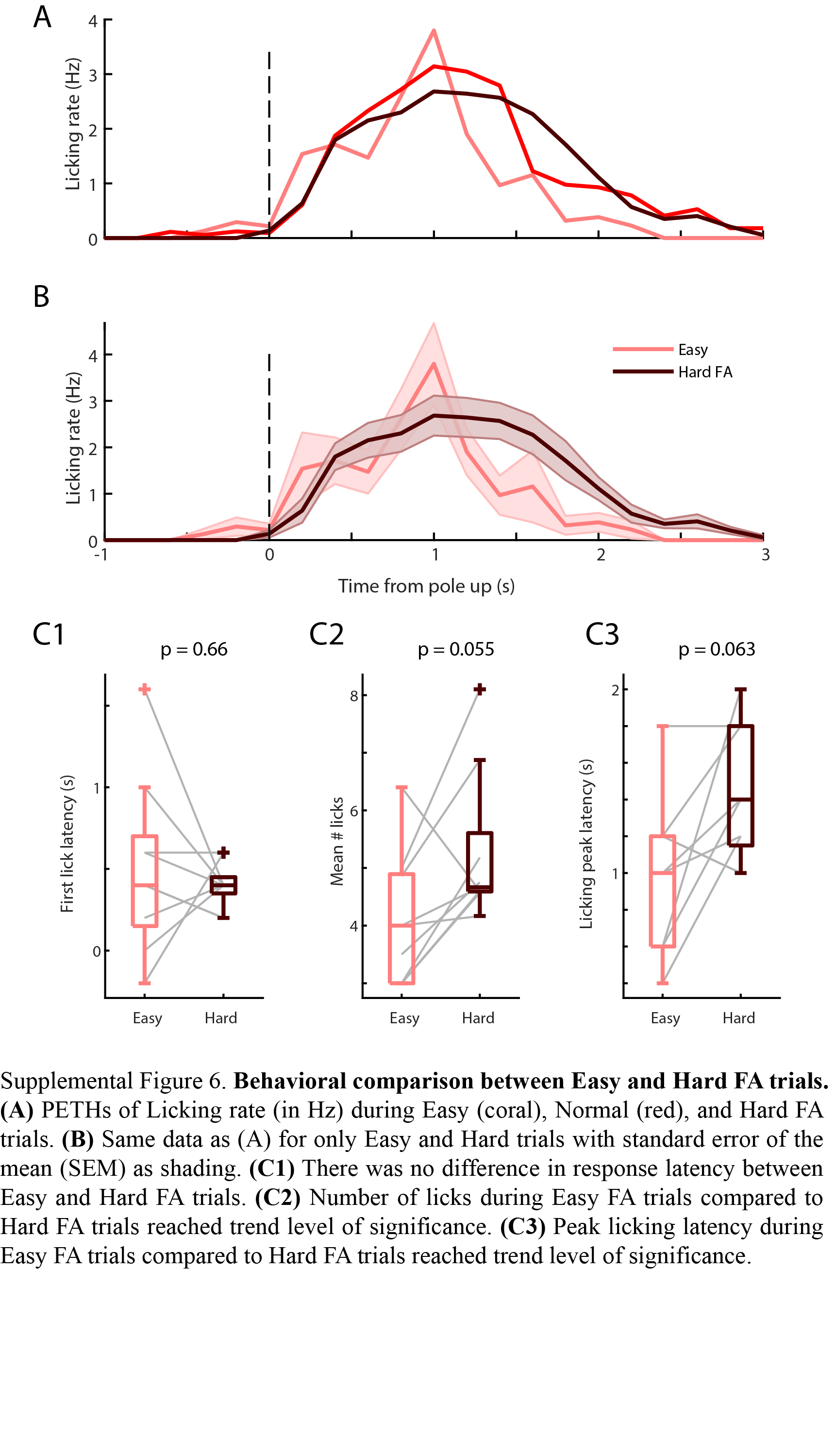

Supplement: Supplementary file 6 [file Image_6.JPEG]

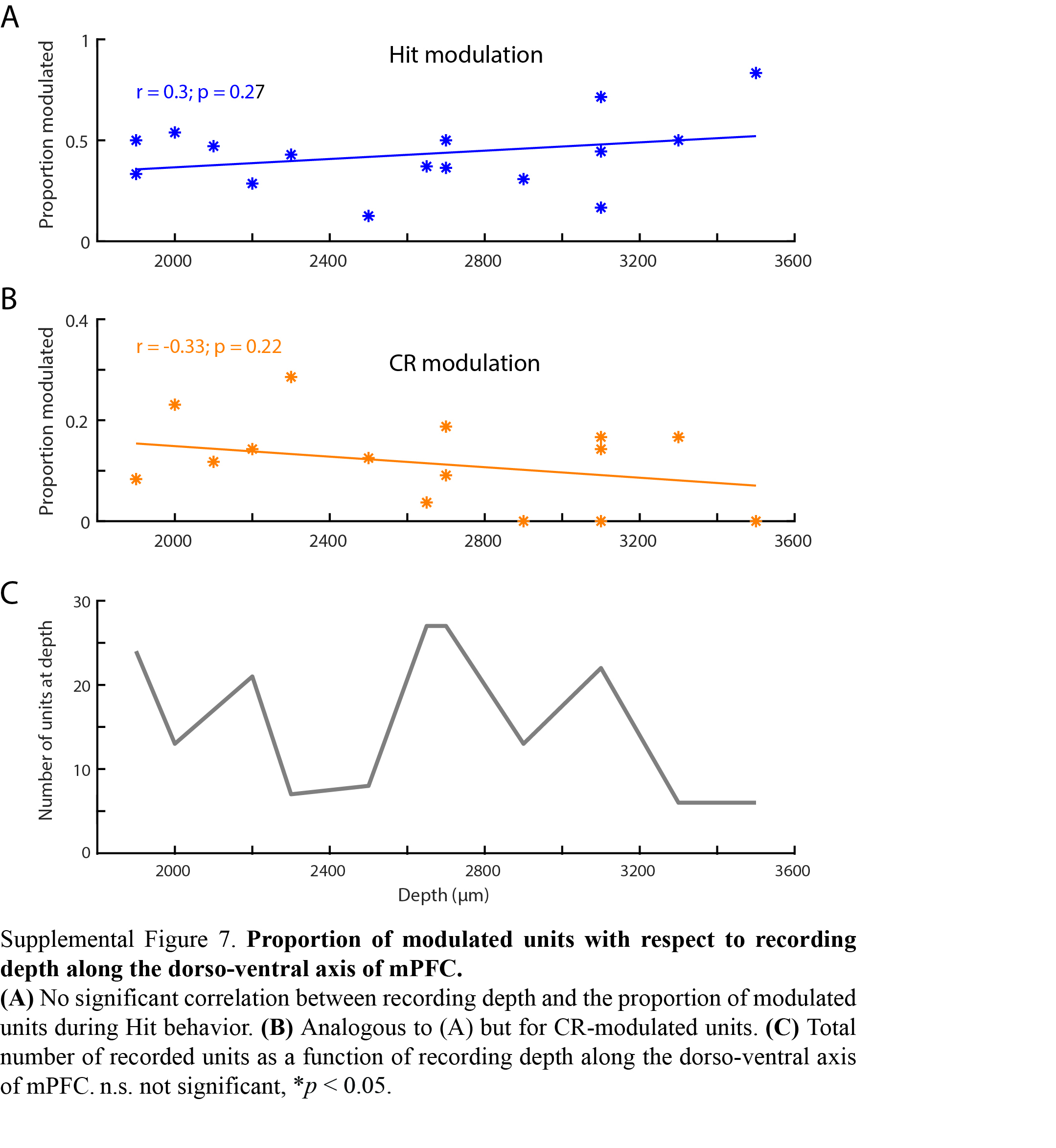

Supplement: Supplementary file 7 [file Image_7.JPEG]
